# Salvage therapy with mitoxantrone, etoposide, bleomycin and dexamethasone for refractory or relapsed aggressive non-Hodgkin’s lymphoma patients with a poor performance status or comorbidity

**DOI:** 10.3892/ol.2014.2517

**Published:** 2014-09-09

**Authors:** XUEDE LIN, XI SHI, WUCHA ZENG, MIN ZHENG, LIMING HUANG

**Affiliations:** Department of Chemotherapy, The First Affiliated Hospital, Fujian Medical University, Fuzhou, Fujian 350005, P.R. China

**Keywords:** mitoxantrone, salvage therapy, non-Hodgkin’s lymphoma, poor performance status, comorbidity

## Abstract

The treatment of refractory or relapsed aggressive non-Hodgkin’s lymphoma (NHL) in patients in a state of poor health is difficult due to their ineligibility to receive intensive salvage chemotherapy. In the present study, 16 refractory or relapsed aggressive NHL patients with a poor performance status or comorbidities were treated with mitoxantrone, etoposide, bleomycin and dexamethasone (MEBD) therapy. The treatment consisted of 10 mg/m^2^ intravenous (IV) mitoxantrone on day 1, 75 mg/m^2^ IV etoposide on days 1–3, 20 mg IV dexamethasone on days 1–4 and 15 mg intramuscular bleomycin on days 1, 4, 8 and 12, every 21 days. The efficacy and toxicity of the regimen were evaluated. The overall response rate was 68.8%, with a complete response rate of 18.8% and a partial response rate of 50.0%. The efficacy of the treatment for B-cell lymphoma was greater than that for T-cell lymphoma. The median progression-free survival time for the patients was 16.7 months and the median overall survival time was 22.4 months. The one-year overall survival rate was 62.5% and the two-year overall survival rate was 43.8%. The most common toxicity symptom was myelosuppression. In conclusion, refractory or relapsed aggressive NHL patients with a poor performance status or comorbidity are eligible for chemotherapy. MEBD therapy is an effective and feasible salvage regimen for NHL patients in a state of poor health.

## Introduction

The majority of aggressive non-Hodgkin’s lymphoma (NHL) cases originate from B cells, with ~10% arising from T cells (1). The standard first-line chemotherapy for the majority of aggressive NHL cases is cyclophosphamide, doxorubicin, vincristine and prednisolone (CHOP) or R-CHOP, a combination of CHOP and rituximab, a monoclonal antibody to cluster of differentiation 20 (2,3). Although the majority of patients with aggressive NHL are responsive to the initial chemotherapy, 40 to 60% either fail to achieve a complete response (CR) following first-line chemotherapy or relapse subsequent to obtaining CR (4).

The current standard treatment strategy for refractory or relapsed NHL is high-dose therapy and autologous stem cell transplantation (HD-ASCT) with curative intent in patients without comorbidities (5,6). However, HD-ASCT is only suitable for fit, young patients who are chemosensitive to salvage chemotherapy. In the absence of hematopoietic stem cell transplantation, the majority of the current treatment strategies for refractory or relapsed NHL are palliative (7–9). The majority of patients are not eligible for ASCT due to refractory disease, age, a poor performance status, comorbidities and other individual reasons (5,10,11). Therefore, alternative salvage approaches have to be employed in these patients. The standard salvage chemotherapy for these NHL patients has not been determined. Prior to the advent of novel chemotherapeutic or targeted agents, the ideal approach for these patients remains as a chemotherapeutic regimen with a high response rate and less toxicity, and containing chemotherapeutic agents that are not cross-resistant to previous therapy.

For refractory or relapsed aggressive NHL patients with a poor performance status or comorbidities, treatment efficacy and quality of life require careful simultaneous consideration. In the present study, the mitoxantrone, etoposide, bleomycin and dexamethasone (MEBD) regimen, which is composed of myelosuppressive (mitoxantrone and etoposide) and non-myelosuppressive (bleomycin and dexamethasone) drugs, was used to treat a group of such patients, and the response rates and toxicities were investigated.

## Patients and methods

### Patients

A retrospective analysis of 16 patients treated in the First Affiliated Hospital, Fujian Medical University (Fuzhou, China) between 2009 and 2012 was conducted. All patients had pathologically confirmed aggressive NHL and had been previously treated with at least one anthracycline-based chemotherapeutic agent. All patients had either an Eastern Cooperative Oncology Group (ECOG) performance status (12) of 2.0–4.0 or comorbidities. Among the patients with comorbidities, one presented with bronchiectasis, one with deep venous thrombosis, two with diabetes and five with chronic hepatitis B infection. Patients with primary central nervous system lymphoma or testicular involvement were not included in the present study. Prior to MEBD chemotherapy, all patients were staged according to the Ann Arbor classification (13), with physical examination, bone marrow biopsy and computed tomography (CT) scans of the neck, chest, abdomen and pelvis. Serum lactate dehydrogenase (LDH) and β2-microglobulin levels were also analyzed. In addition, baseline electrocardiogram (ECG) and ultrasonic cardiogram examinations were performed. The patients were required to have adequate bone marrow, hepatic and renal function, defined as a white blood cell count of ≥3,500/mm^3^, an absolute neutrophil count of ≥1,500/mm^3^, a platelet count of ≥100,000/mm^3^, alanine aminotransferase or aspartate aminotransferase levels <2.0 times the upper normal limit, a bilirubin level of ≤1.5 times the upper normal limit and a serum creatinine level of ≤1.5 times the upper normal limit. This study was approved by the ethics committee of the First Affiliated Hospital, Fujian Medical University.

### Treatment schedule

Once written informed consent had been obtained, all patients received systemic chemotherapy with the MEBD regimen, consisting of 10 mg/m^2^ intravenous (IV) mitoxantrone on day 1, 75 mg/m^2^ IV etoposide on days 1–3, 20 mg IV dexamethasone on days 1–4 and intramuscular 15 mg bleomycin on days 1, 4, 8 and 12, and the cycles were repeated every 21 days. If toxicity occurred, the dose was adjusted according to the physician. If hematological toxicity occurred, prophylactic granulocyte colony-stimulating factor (G-CSF) was used in subsequent cycles. The treatment was continued until either a maximum of six cycles, disease progression, the occurrence of unacceptable toxicity or the decision of the patient to withdraw. During the chemotherapy period, low molecular weight heparin calcium injections (0.3 ml; 3075 AXaIU, twice daily) were provided for the patient with deep venous thrombosis, insulin was administered to the diabetes patients, with monitored blood glucose levels, and entecavir tablets (0.5 mg, daily) were used in the patients with hepatitis B infection for 6 months following chemotherapy, with monitored blood hepatitis B virus DNA concentrations.

### Response and toxicity evaluation

For the response evaluation, CT scans were performed every two cycles of chemotherapy until the end of the treatment and every two months during follow-up. Bone marrow biopsies were performed every two cycles of chemotherapy until the end of the treatment and according to the physician during follow-up. Blood, ECG and ultrasonic cardiogram results were monitored for signs of hematological or cardiac toxicity. Tumor responses, including complete response (CR), partial response (PR), stable disease (SD) and progressive disease (PD) were assessed by the Response Evaluation Criteria In Solid Tumors (14). The overall response rate (ORR) was calculated as the CR plus PR. The progression-free survival (PFS) time was calculated as the time period between the date of MEBD chemotherapy and the date of disease progression. The overall survival (OS) time was calculated as the time period between the initial MEBD treatment and the time-point at which the patient succumbed to the disease. Toxicity was graded by the National Cancer Institute Common Terminology Criteria, version 3.0 (15).

## Results

### Patient characteristics

A total of 16 refractory or relapsed aggressive NHL patients with a poor performance status or comorbidity were treated. The baseline characteristics of the patients and the previous treatments received are summarized in Table I. The median age of the patients was 55 years (range, 35–79 years), and 11 patients were male and five were female. Seven patients had ECOG performance status scores of 2.0–4.0. Nine patients presented with comorbidities. Histologically, 11 patients (68.8%) presented with diffuse large B-cell lymphoma (DLBCL) and five patients (31.2%) with peripheral T-cell lymphoma (PTCL). Two patients (12.5%) were at stage I or II and 14 patients (87.5%) were at stage III or IV. A total of 13 patients (81.3%) had elevated serum LDH levels and all 16 patients (100%) had elevated serum β2-microglobulin levels. With regard to previous chemotherapy, 13 patients (81.3%) had been administered CHOP chemotherapy, two patients (12.5%) had received R-CHOP chemotherapy and one patient (6.25%) had experienced CHOP + etoposide chemotherapy. Among these patients, six (37.5%) had been treated by at least one further regimen in addition to anthracycline-based chemotherapy. Six patients (37.5%) were refractory to previous chemotherapy and 10 patients (62.5%) had relapsed subsequent to previous chemotherapy.

### Treatment response and survival time

Out of the 16 patients, three (18.8%) achieved a CR, eight (50.0%) obtained PR, one (6.2%) exhibited SD and four (25.0%) developed PD. The ORR (CR + PR) was 68.8%. Among the 11 DLBCL patients, a CR was achieved in 18.2% (2/11) and PR in 63.6% (7/11); thus ORR was reached in 81.8% patients (9/11). Among the five PTCL patients, the CR rate was 20.0% (1/5), the PR rate was 20.0% (1/5) and the ORR was therefore 40.0%. The patients with refractory and relapsed aggressive NHL were all responsive to the MEBD chemotherapy. The treatment results are summarized in Table II. The median PFS time was 16.7 months and the median OS time was 22.4 months. The one-year overall survival rate was 62.5% and the two-year overall survival rate was 43.8%. At present, 10 patients remain alive and three of these patients remain with a CR. One patient has remained in CR for 47 months thus far.

### Toxicity

The side-effects of chemotherapy are presented in Table III. The hematological toxicity was severe, with grade 3/4 neutropenia observed in 11 patients (68.8%) and two febrile neutropenia cases (12.5%). Grade 3/4 thrombocytopenia occurred in 18.8% of cases, but only grade 1/2 anemia was observed. The majority of non-hematological toxicity consisted of hepatic dysfunction and gastrointestinal reactions, which were mild and transient. Grade 1/2 interstitial pneumonia occurred in four patients (25.0%). Grade 1 arrhythmia was identified in two patients (12.5%). No renal damage or treatment-related mortality were detected.

## Discussion

As the majority of patients have an incomplete or only temporary response to salvage therapy, the treatment of refractory or relapsed aggressive NHL remains a problem. Salvage chemotherapy regimens normally contain different drugs from those used previously, usually employing non-anthracycline-containing regimens to prevent drug resistance and cumulative toxicity. A number of salvage chemotherapy regimens have been developed to treat refractory or relapsed aggressive NHL. Currently, the majority of the effective salvage approaches use ifosfamide, cytarabine/platinum or gemcitabine. The most commonly used salvage regimens are dexamethasone, cytarabine and cisplatin (DHAP) (16–18), etoposide, methylprednisone, cytarabine and cisplatin (ESHAP) (16,19), carmustine, etoposide, cytarabine and melphalan (mini-BEAM) (20,21), ifosfamide, carboplatin and etoposide (ICE)(18,22,23) and gemcitabine, dexamethasone and cisplatin (GDP) (16,24–26). These regimens have comparable efficacy, resulting in ORRs of 37–68% and complete remission rates of 12 to 37% (19, 21–27). However, the high incidence of hematological toxicity and nephrotoxicity limits the application of these drug regimens in elderly, heavily treated or unfit patients (27).

Mitoxantrone, an anthracenedione antibiotic, exhibits similar clinical activity to the anthracyclines. Mitoxantrone intercalates into DNA through hydrogen bonding, resulting in crosslinks and strand breaks. In addition, mitoxantrone interferes with RNA and is a potent inhibitor of topoisomerase II, an enzyme responsible for uncoiling and repairing of damaged DNA (28,29). In preclinical lymphoma models, the potent activity of mitoxantrone has been demonstrated, with the drug appearing to be clinically active against follicular and aggressive lymphomas (39–32). However, controversy remains with regard to the superiority of mitoxantrone or anthracyclines in the treatment of elderly NHL patients (33,34). It has been hypothesized to that mitoxantrone retains the antineoplastic effects of the anthracyclines, but with less potential for cardiotoxicity, as mitoxantrone does not have the amino sugar of doxorubicin or the characteristic ring structure of the classical anthracyclines (28,35). Mitoxantrone has only partial cross-resistance with anthracyclines, such as Adriamycin (36,37), and the efficacy of mitoxantrone appears to be less affected by multidrug resistance than Adriamycin or etoposide (36,38,39). In theory, anthracycline-resistant tumors are sensitive to mitoxantrone, and mitoxantrone may exert a synergistic effect with etoposide. The first-line use of etoposide, mitoxantrone, cyclophosphamide, vincristine, prednisolone and bleomycin (VNCOP-B) has produced an 83% ORR and a 58% CR rate in elderly patients with aggressive NHL (40). However, in this study, grade 4 neutropenia was shown to occur in 29% patients on this regimen. The combined use of etoposide, mitoxantrone and prednisone has achieved a 38% ORR among refractory or relapsed NHL patients, with relatively low toxicity (41). Therefore, in the present study, these regimens were modified and a novel combination chemotherapy regimen, MEBD, was developed, which comprises myelosuppressive (mitoxantrone and etoposide) and non-myelosuppressive (bleomycin and dexamethasone) drugs in order to increase efficacy and reduce toxicity.

In the present study, MEBD treatment, used as a salvage chemotherapeutic regimen in patients with aggressive NHL, achieved a 68.8% ORR and an 18.8% CR rate, with a median PFS of 16.7 months and a median OS of 22.4 months. Certain patients achieved long-term survival. The preliminary results appear comparable with those from patients treated with the aforementioned intensive salvage regimens, such as DHAP, ESHAP, mini-BEAM, ICE and GDP. Furthermore, the results of the present study were obtained from refractory or relapsed patients with poor health conditions, with 87.5% patients at stage III or IV. The results demonstrate that MEBD is an efficacious salvage regimen for patients with aggressive NHL who are in a state of poor health.

MEBD therapy appeared to have greater efficacy in B-cell lymphoma than in T-cell lymphoma. For the DLBCL patients, the CR rate was 18.2% and the PR rate was 63.6%, thus the ORR was 81.8%. Among the PTCL patients, the CR was 20.0% and the PR was 20.0%, therefore the ORR was 40.0%. The refractory and relapsed NHL patients who had experienced anthracycline-based chemotherapy were observed to respond to MEBD treatment. This demonstrates that mitoxantrone-containing regimens have little cross-resistance to anthracyclines. As the present study was limited by the low number of patients, further large group studies are required in order to draw definite conclusions.

Due to the poor health conditions of the patients in the present study, hematological toxicity remained severe even if a moderate dosage of mitoxantrone and etoposide was used and the non-myelosuppressive agents bleomycin and dexamethasone were applied. Grade 3/4 neutropenia was identified in 68.8% of patients and grade 3/4 thrombocytopenia was observed in 18.8% of cases. Drug doses had to be adjusted for these patients, with prophylactic G-CSF used in the following cycles of chemotherapy. Cardiotoxicity was mild even if all patients had previously received anthracycline-based chemotherapy. Only two patients (12.5%) presented with grade 1 arrhythmia. Hepatic dysfunction was also mild; five patients (31.3%) exhibited grade 1 and one patient (6.3%) exhibited grade 2 transient toxicity. The gastrointestinal reactions that were detected were not severe and were controllable. Interstitial pneumonia occurred in 25.0% of patients due to the use of bleomycin. Although the interstitial pneumonia identified was classified as grade 1/2 and curable, the toxicity impeded the continuation of MEBD chemotherapy. The bleomycin dose modification required to balance the treatment efficacy and the lung toxicity requires further investigation. In VNCOP-B treatment, 10 mg/m^2^ IV bleomycin was used only once every four weeks and no interstitial pneumonia was detected (40).

In conclusion, refractory or relapsed aggressive NHL patients with a poor performance status or comorbidity remain eligible for chemotherapy. MEBD is an effective and feasible salvage regimen with long-term survival efficacy for patients in a state of poor health. The most severe toxicity symptom is myelosuppression and prophylactic measures are recommended to prevent hematological toxicity.

## Figures and Tables

**Table I:** Patient characteristics.

Characteristic	Value
Median age (range), years	55 (35–79)
Gender, n	
Male	11
Female	5
ECOG performance status, n	
0–1	9
2–4	7
Stage, n	
I/II	2
III/IV	14
B-symptoms, n	9
LDH level	
Elevated	13
Normal	3
β2-microglobulin level, n	
Elevated	16
Normal	0
Bulky mass, n	3
Bone marrow involvement, n	1
Disease status, n	
Relapsed	10
Refractory	6
Previous chemotherapy, n	
CHOP	13
R-CHOP	2
CHOP-E	1
≥2 regimens	6
Previous high-dose chemotherapy, n	2
Previous radiotherapy, n	2
NHL subtype, n	
Diffuse large B-cell lymphoma	11
Peripheral T-cell lymphoma	5

ECOG, Eastern Cooperative Oncology Group; LDH, lactate dehydrogenase; CHOP, cyclophosphamide, doxorubicin, vincristine and prednisolone; CHOP-E, CHOP + etoposide; NHL, non-Hodgkin’s lymphoma.

**Table II:** Patient responses to MEBD.

	Response, n (%)			
	
Patient characteristic	CR	PR	SD	PD
All patients	3 (18.8)	8 (50.0)	1 (6.3)	4 (25.0)
Histological type				
B-Cell	2 (18.2)	7 (63.6)	0 (0.0)	2 (18.2)
T-Cell	1 (20.0)	1 (20.0)	1 (20.0)	2 (40.0)
Pre-treatment status				
Relapsed	3 (30.0)	5 (50.0)	0 (0.0)	2 (20.0)
Refractory	0 (0.0)	3 (50.0)	1 (16.7)	2 (33.3)

MEBD, mitoxantrone, etoposide, bleomycin and dexamethasone; CR, complete response; PR, partial response; SD, stable disease; PD, progressive disease.

**Table III:** Toxicity.

	Number of patients (%)			
	
Adverse effect	Grade 1	Grade 2	Grade 3	Grade 4
Leukopenia	3 (18.8)	2 (12.5)	4 (25.0)	7 (43.8)
Neutropenia	2 (12.5)	3 (18.8)	5 (31.3)	6 (37.5)
Anemia	6 (37.5)	3 (18.8)	0 (0.0)	0 (0.0)
Thrombocytopenia	8 (50.0)	5 (31.3)	2 (12.5)	1 (6.3)
Febrile neutropenia	0 (0.0)	0 (0.0)	2 (12.5)	0 (0.0)
AST/ALT elevation	5 (31.3)	1 (6.3)	0 (0.0)	0 (0.0)
Bilirubin elevation	2 (12.5)	0 (0.0)	0 (0.0)	0 (0.0)
Mucositis	0 (0.0)	1 (6.3)	0 (0.0)	0 (0.0)
Nausea	6 (37.5)	3 (13.8)	0 (0.0)	0 (0.0)
Vomiting	4 (25.0)	0 (0.0)	0 (0.0)	0 (0.0)
Constipation	7 (43.8)	2 (12.5)	0 (0.0)	0 (0.0)
Arrhythmia	2 (12.5)	0 (0.0)	0 (0.0)	0 (0.0)
Interstitial pneumonia	1 (6.3)	3 (18.8)	0 (0.0)	0 (0.0)

AST, aspartate aminotransferase; ALT, alanine aminotransferase.
